# Protocol for the quantification of melatonin and AFMK in cerebrospinal fluid by LC-MS/MS

**DOI:** 10.1016/j.xpro.2025.104145

**Published:** 2025-10-22

**Authors:** Francisco Artime-Naveda, David Hevia-Sánchez, Adriana González-Gago, Pablo Rodríguez-González, Anna Zawadzka, Rosa M. Sainz, Juan C. Mayo

**Affiliations:** 1 Departamento de Morfología y Biología Celular, Faculty of Medicine, University of Oviedo, Julián Clavería, 6, 33006 Oviedo, Spain; 2 Instituto Universitario de Oncología del Principado de Asturias (IUOPA), University of Oviedo, Fernando Bongera s/n, 33006 Oviedo, Spain; 3 Instituto de Investigación Sanitaria del Principado de Asturias (ISPA), Avda. Hospital Universitario s/n, 33011 Oviedo, Spain; 4 Departamento de Química Física y Analítica, Faculty of Chemistry, University of Oviedo, Avda. Julián Clavería, 8, 33006 Oviedo, Spain; 5 Laboratory of Natural Products Chemistry, Faculty of Chemistry, University of Warsaw, Pasteura Str 1, 02-093 Warsaw, Poland

**Keywords:** Cell Biology, Mass Cytometry, Health Sciences, Neuroscience

## Abstract

Melatonin and its oxidant derivative, N-acetyl-N-formyl-5-methoxykynuramine (AFMK), are indolamines present in higher organisms, which are related to light-darkness circadian rhythms and redox physiology. Here, we present a protocol for the quantification of both in cerebrospinal fluid (CSF). We describe steps for standardization by isotopic dilution or alternatively by internal standard 5-methoxytryptophol (5-MT). We then detail procedures for quantification by using liquid chromatography coupled to tandem mass spectrometry.

For complete details on the use and execution of this protocol, please refer to Artime-Naveda et al.^1^

## Highlights

• Steps to determine melatonin and AFMK by isotopic dilution and internal standardization
• Steps for liquid chromatographic separation of melatonin and derivatives
• Details on CSF sample extraction and preparation for mass spectrometry analysis

## Abstract (editor highlights)

Publisher’s note: Undertaking any experimental protocol requires adherence to local institutional guidelines for laboratory safety and ethics.

## Graphical abstract

**Figure:**
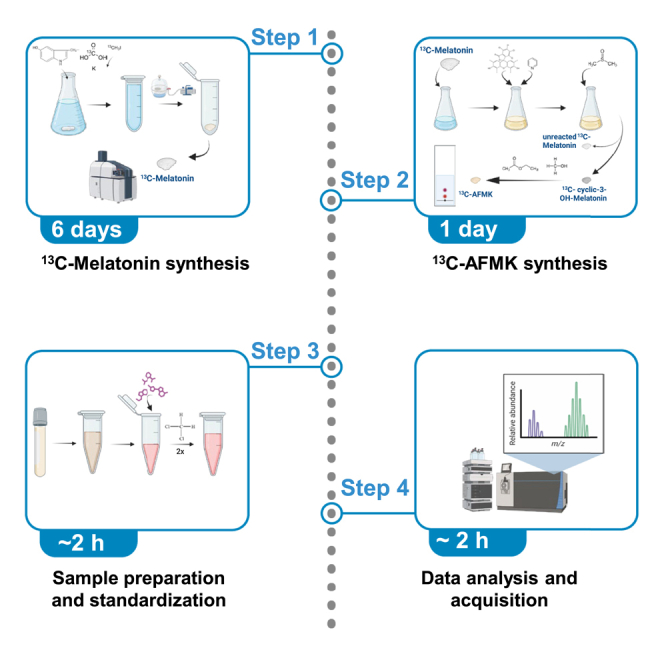

## Before you begin

Melatonin (N-Acetyl-5-methoxytryptamine, C_13_H_16_N_2_O_2_, M.W 232.28) is a ubiquitous molecule present most cell organisms, from unicellular to plants and animals. This indolamine can interact directly with a multitude of biological targets controlling the physiology and homeostasis of most living beings, with a role as an authentic ‘chemical expression’ of darkness.^2^ One of the main functions of melatonin is to regulate the circadian cycles of light and darkness. Considering the relationship between sleep rhythm disorders and neurological origin, the therapeutic potential of melatonin as a neuroprotective agent has been observed against Parkinson’s disease, amyotrophic lateral sclerosis, Huntington’s disease and brain trauma.^3^^,^^4^^,^^5^ Antioxidant properties of melatonin have been widely demonstrated. Antioxidant reactions of the indolamine can trigger formation of different metabolites which also display antioxidant properties, including AFMK (N1-acetyl-N2-formyl-5-methoxykynuramine, C_13_H_16_N_2_O_4_).^6^ The close relationship between oxidative stress, melatonin and its derivatives, and the neurodegenerative processes highlights the importance of developing analytical methodologies that allow for their accurate determination and their correlation with the clinical outcome.

To date, multiple methods have been used for melatonin quantification, including RIA and more recently ELISA. Implementation of LC-MS/MS devices in most analytical facilities, together with their well-known higher sensitivity, have become the preferred and most commonly used analytical method for the determination of the indolamine. Here we describe the determination of melatonin and AFMK in cerebrospinal fluid (CSF) using LC/MS-MS methodology based on a triple quadrupole detector, constituting a suitable alternative for the determination of melatonin in different biological substrates using similar, less sensitive methodologies.^7^^,^^8^^,^^9^ Furthermore, triple quadrupole detector due to its versatility, precision and sensitivity allows the detection and quantification of a low number of metabolites at the same run. However, other types of detectors could be used if they present sensitivity and resolution specifications similar to those related to the detector used in this work. Due to the clinical relevance of melatonin in different neurodegenerative processes and its relationship with aging,^10^ in this study, a methodology for the determination of melatonin and its derivative AFMK in cerebrospinal fluids with isotopic dilution enrichment will be developed.

The standardization protocol used is based on the addition of an internal standard to correct analyte losses in extraction and matrix effects. Due to the identical chemical behavior of the different isotopologues of a certain compound, the addition of isotopically labeled standards ^13^C in one carbon atom presented in this protocol constitutes an interesting alternative to the use of conventional internal patterns, in this case 5-MT, which will be presented as an alternative, in case the ^13^C standards are not available for the users. The application of isotopic dilution standardization model considerably reduces sample preparation and matrix effects, reducing the interindividual deviation and allowing for a more precise quantification in determination.^11^

In this protocol, CSFs samples processing and the application of the standardization model by isotopic dilution or internal standards (5-methoxytryptophol, 5-MT, C_11_H_13_NO_2_) for determination of both melatonin and AFMK will be explained in detail.

For complete details on the use of conventional internal standardization and isotopic dilution protocol in melatonin determination refer to Fernandez A et al.^12^

### Innovation

The application of an isotopic dilution standardization method described in this protocol allows the determination and quantification of melatonin and AFMK in cerebrospinal fluid samples without the need for any external calibration, in addition to being able to determine both metabolites in a single measurement by means of separation by liquid chromatography and detection by triple quadrupole mass spectrometry.

### Institutional permissions

Patient’s CSF sample collection was previously approved by the Ethical Committee Board (Internal Reference #2023.578 of the Ethical Committee Board at the ‘Hospital Universitario Central de Asturias’, HUCA), following the ethical principles for Medical Research involving human subjects adopted by the ‘World Medical Association Declaration of Helsinki. For the purpose of the study, the committee approved the waiver of informed consent, since a small fraction of samples for diagnostic assessment were used. Samples were extracted by lumbar puncture and once the necessary volume for diagnosis was employed, aliquots of each sample were stored at −80°C.

Note for readers/researchers: Researchers interested in following the present protocol using patients’ samples should be aware that the necessary ethical approvals should be obtained previously, according to their local and institutional regulations under the supervision of the corresponding Ethical Committees.

### Preparation one: Organic synthesis of ^13^C_1_ melatonin and ^13^C_1_ AFMK

**Timing:**^**13**^**C**_**1**_**Melatonin synthesis, 6 days;**^**13**^**C**_**1**_**AFMK synthesis, 1 day**

1. ^13^C_1_ Melatonin synthesis (for further details on the synthesis, please refer to Artime-Naveda et al^1^ and Fernández et al^12^).
  a. Stir for 20 min a mixture of N-Acetyl-5-hydroxytryptamine (200 mg, 0.92 mmol), potassium carbonate (381 mg, 2.76 mmol) and 18-Crown-6 (24 mg, 0.09 mmol) in 10 mL of acetone at 20°C.
  b. Add Iodomethane-^13^C (1.84 mmol) and stir at 15°C–20°C in closed sealing vial for 5 days.
  c. Remove the solvent in vacuo.
    i. Dilute the residue with 50 ml of ultrapure (milli-Q or HPLC-grade) water.
    ii. Extract three times with chloroform (3∗50 ml).
  d. Wash the extracted organic phases with 50 ml of brine.
    i. Dry over magnesium sulfate (1 g) for 30 min.
    ii. Separate the magnesium sulfate by filtering through a filter paper.
    iii. Concentrate the filtrate under reduced pressure.
  e. Purify (duration about 2h) the residue by column chromatography on silica gel 60 (230-400 mesh), (eluent: Chloroform/Methanol 99:1 to obtain the target compound as white solid.

***Note:*** Product structure was confirmed by ^1^H NMR, ^13^C NMR and HR MS analysis (Data S1: Melatonin and AFMK ^13^C_1_ nuclear magnetic resonance spectrum). Expected synthesis yield roughly 85%.

2. ^13^C_1_ AFMK synthesis (for further details on the synthesis, please refer to Artime-Naveda et al^1^ and Siwicka et al^13^):
  a. Dissolve 220 mg of previously synthesized ^13^C_1_ melatonin in 200 ml of dichloromethane and methanol mixture (2:1, v/v).
    ***Note:*** Reaction was carried out in an efficiently functioning fume hood.
  b. Add 1 mL of dry pyridine and 40 mg of Rose Bengal dye and immerse the flask in ethanol/dry ice bath.
  c. Irradiate the mixture with 400 W halogen lamp for 10 h with vigorous stirring in an oxygen-rich atmosphere (oxygen balloon connected).
    ***Note:*** Conduct the reaction in an ethanol/dry bath.
  d. Replace the oxygen-rich atmosphere with inert gas (either argon or nitrogen, technical grade, can be employed) and add 2 ml of dimethyl sulphide.
    ***Note:*** The mixture was allowed to reach 15°C–25°C for 10 h.
  e. Evaporate the residue in vacuo and purify by gravity column chromatography (column size: 10 mm diameter, length 200 mm) on neutral alumina 60 (activity III).
    ***Note:*** Elution with chloroform allowed the recovery of unreacted ^13^C-melatonin (110 mg).
  f. Purify again the main product-free fraction by column chromatography on preparative silica gel with 2% (v/v) methanol in ethyl acetate to give 30 mg of ^13^C AFMK.
    ***Note:*** Subsequent elution with 3% (v/v) methanol in dichloromethane gave the ^13^C cyclic 3-hydroxymelatonin as an amorphous solid in 70 mg yield.
    ***Note:*** TLC analysis on alumina in dichloromethane: methanol 97:3 system indicated the absence of main product of this reaction - ^13^C-cyclic 3-hydroxymelatonin.

### Preparation two: Melatonin, AFMK, 5-MT, and ^13^C_1_-enriched standard preparation

**Timing: 2 h**

3. Prepare standards and enriched ^13^C standards solutions.

**Table:** Stock solutions

Reagent	Final concentration	Amount	Acetonitrile
^13^C_1_ Melatonin standard	250 μg/g	1 mg	5 ml
Melatonin standard	250 μg/g	1 mg	5 ml
^13^C_1_ AFMK standard	250 μg/g	1 mg	5 ml
AFMK standard	250 μg/g	1 mg	5 ml
5-Methoxytryptophol	2500 μg/g	10 mg	5 ml

***Note:*** Weigh the mass of standard and the mass of solvent to perform the exact calculation of the final concentration in ppm (μg/g). Prepare separately.

***Note:*** Aliquot and store at −80°C and in dark up to 1 year.

**Table:** External standards stock solutions

Reagent (stock solutions)	Final concentration	Amount	Acetonitrile
Melatonin standard (250 ppm)	1 ppm	20 μl	4980 μl
AFMK standard (250 ppm)	5 ppm	100 μl	4900 μl
5-Methoxytryptophol (2500 ppm)	1000 ppm	2000 μl	3000 μl

***Note:*** Weigh the mass of standard and the mass of solvent to perform the exact calculation of the final concentration in ppm (μg/g). Prepare separately.

***Note:*** Aliquot and store at −80°C and in dark up to 1 year.

**Table:** Internal standards stock solutions

Reagent (stock solutions)	Final concentration	Amount	Acetonitrile
^13^C_1_ Melatonin standard (250 ppm)	1 ppm	20 μl	4980 μl
^13^C_1_ AFMK standard (250 ppm)	1 ppm	20 μl	4980 μl
5-Methoxytryptophol (2500 ppm)	50 ppm	100 μl	4900 μl

***Note:*** Weigh the mass of standard and the mass of solvent to perform the exact calculation of the final concentration in ppm (μg/g). Prepare separately.

***Note:*** Aliquot and store at −80°C and in dark up to 1 year.

### Preparation three: Preparation of ^13^C_1_ melatonin and ^13^C_1_ AFMK standards for isotopic abundance determination

**Timing: 30 min**

4. Prepare separately 1 ppm melatonin, ^13^C_1_ melatonin, AFMK and ^13^C_1_ AFMK standard in water: ACN (94.4:5.6).

### Preparation four: Determining the experimental relative isotopic abundance of ^13^C_1_ melatonin and ^13^C_1_ AFMK standards

**Timing: 1 h and 30 min**

5. Measure the areas of the transitions (precursor ion fragmentation to product ion) described in materials and equipment set up, MRM transitions for ^13^C_1_ melatonin, ^13^C_1_ AFMK and natural melatonin and AFMK.
6. Determine the relative isotopic abundance for each transition by summing all the areas and dividing each by the total sum.

***Note:*** Isotopic abundance of synthesized ^13^C_1_ Melatonin and ^13^C_1_ AFMK is required to be experimentally determined due to the theoretical lack of knowledge of the percentage of enrichment after organic synthesis.

## Key resources table

**Table:** 

REAGENT or RESOURCE	SOURCE	IDENTIFIER
**Biological samples**		

Cerebrospinal fluids (CSF)	Hospital Universitario Central de Asturias, HUCA	Internal Reference #2023.578 of the Ethical Committee Board

**Chemicals, peptides, and recombinant proteins**		

Acetone	Sigma-Aldrich	179124
Dimethyl sulfide	Sigma-Aldrich	8.20833
Ethanol	Sigma-Aldrich	459844
Ethyl acetate	Sigma-Aldrich	319902
Methanol	Sigma-Aldrich	179337
Chloroform	Sigma-Aldrich	319988
Magnesium sulfate	Sigma-Aldrich	M2643
Alumina gel	Merck	1.01077
Silica gel	Merck	717185
Melatonin for synthesis	Merck	Ref. 8.14537
5-methoxytryptophol	MedChemExpress (MCE)	Ref HY-113440
AFMK	Cayman Chemical	Item No. 10005254
Potassium carbonate	Sigma-Aldrich	Ref. 367877
Piridine	Thermo Fisher scientific	Ref. 339420025
^13^C_1_ Melatonin	Organic Synthesis	Organic synthesis of 13C1 melatonin and 13C1 AFMK section
^13^C_1_ AFMK	Organic Synthesis	Organic synthesis of 13C1 melatonin and 13C1 AFMK section
Acetonitrile Optima Grade LC/MS	Thermo Fisher Scientific	Ref A955-212
Formic acid Optima LC/MS	Fisher Chemical	Product code A117-50
Ultra-pure water	Elga Labwater	Purelab Flex 3
Dichloromethane	Sigma-Aldrich	Ref. 32222
N-Acetyl-5-hydroxytriptamine > 98%	TCI	Ref A1277
18-Crown-6 > 98%	Fisher Scientific	Ref. 15640870
Iodomethane*-*^13^C 99 atom% ^13^C, 99%	Sigma-Aldrich	Ref. 277185
Rose Bengal > 96% (HPLC), Dye content 95%	Sigma-Aldrich	Ref. 330000
Quantitative filter paper, circles X100 Grade 388, black dot 110 mm	Ahlstrom-Munksjo Munktell Product code 16384968	Product code 16384968
Wheaton Sample Vials with Wheaton Black phenolic screw caps	Zinsser Analytic	Ref. 224831 Ref. 40418
Halogen Bulb R7S 400v Win Outdoor halogen fixture	Bellight Lena Sp. Z o.o.	Product code 17159334 Product code ZT-500

**Deposited data**		

Mass spectrometry and chromatography settings	Fernández et al.^12^	2.3.5. Chromatographic separation of the samples 2.2. Instrumentation https://doi.org/10.1016/j.chroma.2021.462752
CSF samples melatonin and AFMK results	Artime-Naveda et al.^1^	Figure 5, https://doi.org/10.1016/j.heliyon.2025.e41841

**Software and algorithms**		

Agilent Mass Hunter workstation data acquisition	Agilent	Ref G3337AA
Agilent MassHunter Quantitative Analysis 10.1	Agilent	Ref G3337AA
Agilent MassHunter Qualitative Analysis	Agilent	Ref G3337AA

**Other**		

Chromatography column	Agilent	Zorbax RRHD Eclipse Plus C18 (2.1 × 50 mm, 1.8 μm, 95 Å pore size) Ref. 959757-902
Evaporator (Centrivap Concentrator)	LABCONCO	S/N: 101234529 C
LC_MS tandem equipment	Agilent	Triple quadrupole mass spectrometer Agilent 6460 Agilent 1290 Infinity 2D-LC system

## Materials and equipment

**Table:** External calibration stock

Reagent (external standards stock solutions)	Final concentration	Amount
Melatonin standard 1 ppm	1 ppb	5 μL
AFMK standard 5 ppm	50 ppb	50 μL
5-MT 1000 ppm	10000 ppb	50 μL
Water: ACN (94.4:5.6)	N/A	4895 μL

**Table:** Calibration standard 1

Reagent	Final concentration	Amount
External calibration stock	Mel (ppb): 0.01 5-MT (ppb): 100 AFMK (ppb): 0.5	50 μL
Water: ACN (94.4:5.6)	N/A	4950 μL
Total	N/A	5000 μL

**Table:** Calibration standard 2

Reagent	Final concentration	Amount
External calibration stock	Mel (ppb): 0.05 5-MT (ppb): 500 AFMK (ppb): 2.5	250 μL
Water 5,6 % ACN	N/A	4750 μL
Total	N/A	5000 μL

**Table:** Calibration standard 3

Reagent	Final concentration	Amount
External calibration stock	Mel (ppb): 0.1 5-MT (ppb): 1000 AFMK (ppb): 5	500 μl
Water 5,6 % ACN	N/A	4500 μl
Total	N/A	5000 μl

**Table:** Calibration standard 4

Reagent	Final concentration	Amount
External calibration stock	Mel (ppb): 0.25 5-MT (ppb): 2500 AFMK (ppb): 12.5	1250 μl
Water 5,6 % ACN	N/A	3750 μl
Total	N/A	5000 μl

**Table:** Calibration standard 5

Reagent	Final concentration	Amount
External calibration stock	Mel (ppb): 0.5 5-MT (ppb): 5000 AFMK (ppb): 25	2500 μl
*Water 5,6 % ACN*	N/A	2500 μl
Total	N/A	5000 μl

**Table:** Calibration standard 6

Reagent	Final concentration	Amount
External calibration stock	Mel (ppb): 1 5-MT (ppb): 10000 AFMK (ppb): 50	N/A
Water 5,6 % ACN	N/A	N/A
Total	N/A	N/A

***Note:*** Weigh the mass of standard and the mass of solvent to perform the exact calculation of the final concentration in ppb (ng/g).

***Note:*** Store at −80°C and in darkness for up to a year.

**Table:** Electrospray working conditions

Parameters	
Gas temperature (°C)	250
Gas flow (L/min)	7
Capillary voltage (V)	3500
Sheath gas flow (L/min)	12
Fragmentor voltage (V)	135
Nozzle voltage (V)	0
Nebulizer pressure (psi)	30

**Table:** Chromatographic separation

Time (min)	A (%) water 0.1 % formic acid	B (%) Acetonitrile	Flow (ml/min)
0	94.4	5.6	0.4
3	94.4	5.6	0.4
8	78	22	0.4
12	74	26	0.4
14	30	70	0.4
16	94.4	5.6	0.4

***Note:*** Chromatographic separation at RT on a Zorbax RRHD Eclipse Plus C18. Needle wash not applicable. Sample injection volume 5 μL.

**Table:** SRM transitions

Compound	Precursor ion	m/z	Product ion	m/z	Collision energy (eV)
Melatonin	C_13_H_17_N_2_O_2_	233.1	C_11_H_12_NO	174.1	9
AFMK	C_13_H_17_N_2_O_4_	265.1	C_10_H_12_NO_2_	178.1	11
5-MT	C_11_H_14_NO_2_	192.1	C_11_H_12_NO	174.1	13

***Note:*** Samples diluted in water 94.4%; CAN 5.6%.

**Table:** MRM transitions

Compound	Transitions (m/z)	Collision energy (eV)	Dwell time (ms)	Fragmentor voltage
Melatonin	233.1 → 174.1 234.1 → 175.1 235.1 → 176.1 236.1 → 177.1	9	20	100 V
AFMK	265.1 → 178.1 266.1 → 179.1 267.1 → 180.1 268.1 → 181.1	11	20	100 V

## Step-by-step method details

### Sample standardization and melatonin and AFMK extraction

**Timing: 2 h**

This step consists of melatonin and AFMK extraction applying a liquid-liquid extraction strategy and the standardization of the sample by adding isotopically labeled standards or the internal standard 5-methoxytryptofol prior extraction.

1. Preparation of the labeled standards and addition to CSF samples:
  a. Thaw 1 ppm internal standard ^13^C_1_ melatonin and ^13^C_1_ AFMK solution.
    ***Note:*** Thaw in ice under controlled temperature.
    i. Check that the standards are completely thawed.
    ii. Maintain in ice until working solutions are prepared (0.1 ppb ^13^C_1_ melatonin and 0.5 ppb ^13^C_1_ AFMK respectively).
  b. Thaw CSF samples (100 μL).
    i. Transfer them to a new 1.5 ml tube.
    ii. Weigh them on a precision balance.
  c. Add 30 μl ^13^C_1_ melatonin working solution.
    i. Weigh.
    ii. Add ^13^C_1_ AFMK working solution and weigh again without tare.
      ***Alternatives:*** Thaw 50 ppm internal standard 5-MT, dilute to working solution (500 ppb), add 30 μL of 5-MT working solution and weigh.
      ***Note:*** Weighing process. First, tare the empty tube, add samples and weigh, next add internal standard (^13^C_1_ Mel) (without tare) and weigh. Finally, add ^13^C_1_ AFMK without tare. The value of each weight is noted, and the weight of each added standard is calculated by difference.
2. Liquid-Liquid extraction:
  a. Add 500 μl of dichloromethane (DCM) over the CSF 100 μl sample.
    i. Vortex for 10 min.
  b. Centrifuge the samples at 10,000 g for 5 min at 4°C.
    i. Collect the lower organic phase.
    ii. Transfer the lower organic phase to a fresh tube.
  c. Repeat steps 2a and 2b.
  d. Evaporate completely the samples under speed vacuum at 40°C for 40 min.

**Pause point:** Store the extracted CSF samples at −80°C until further processing up to 1 month.

### LC-MS/MS analysis and data acquisition

**Timing: 20 min per sample and 30 min for data acquisition**

This step consists of processing the extracted samples in the LC-MS/MS system and the subsequent acquisition of data.

3. LC-MS/MS setting and samples measurements:
  a. Reconstitute the samples in 50 μl water: ACN (94.4:5.6) and weigh the added volume.
  b. Transfer the reconstituted samples into a vial with insert and introduce them into the automatic injection module.
  c. Prepare freshly mobile phase A: Water 0.1% formic acid pH=3.5. Mobile phase B: ACN.
    ***Note:*** Check pH by measuring with a pH meter. If it is necessary, adjust to final pH adding formic acid.
  d. Equilibrate the column under initial chromatography conditions (Water, 5.6 % ACN, 0.1 % formic acid).
    ***Note:*** Equilibration time will be required for column pressure stabilization.
    i. Check for the absence of pressure fluctuations.
  e. Generate the sequence and start the run.
    i. Firstly, run mobile phase as a blank. then calibration standards (only in 5-MT standardization method) and finally samples.
4. Data acquisition:
  a. Open the data files in Agilent MassHunter Quantitative Analysis 10.1 program a new batch with all the files in the sequence to be analyzed.
  b. Configure the analysis method based on calibration standard 5.
    i. Select the quantification transitions for melatonin and AFMK (MRM transitions, materials and equipment setup).
      ***Alternatives:*** Select the quantifications transitions for 5-MT (SRM transitions, materials and equipment setup).
  c. Apply the method created to integrate the areas.
  d. Export the data table in Excel.

### Data processing and concentration calculation

**Timing: 1 h**

This step consists of processing the acquired data and calculation of the melatonin and AFMK concentration in the samples by isotopic dilution protocol or standardization by internal standard 5-MT.

5. Metabolites sample concentration by isotopic dilution.
  a. Determine the experimental relative abundance of the samples of each isotopologue by dividing the area of each transition by the total sum of the area of all the transitions.
  b. Apply the Excel function LINEAR ESTIMATION to the experimental relative abundance values and the values of the previously calculated natural and enriched isotopic abundances.
    Record the linear contribution values for the natural and enriched compounds (molar fractions).
  c. Calculate R by the quotient between the natural and enriched molar fraction.
  d. Apply the following formula to calculate the natural compound concentration:
    Natural conc. = R∗(g ^13^C standard/g sample) ∗ (MW natural/MW ^13^C standard) ∗ (Conc. ^13^C standard).
    R = natural molar fraction / enriched ^13^C molar fraction; MW (molecular weight); g (weight).
6. Metabolites sample concentration by internal standardization (5-MT).
  a. Calculate the concentration of Melatonin, AFMK and 5-MT from the areas obtained for each compound.
  b. Multiply the concentration of the three compounds by the weight of the reconstituted sample and then divide by the initial weight.
  c. Determine the percent recovery for 5-methoxytryptofol by comparing the experimental concentration to the theoretical concentration expected for total recovery.
  d. Apply the recovery percentage to the melatonin and AFMK concentrations to obtain the final concentration.

***Note:*** Apply data processing 5 (isotopic dilution) or 6 (internal standardization 5-MT) in function of the internal standardization protocol chosen.

## Expected outcomes

The analysis of the relative isotopic abundance of natural compounds and those isotopically enriched in one ^13^C atom should provide similar abundances to those presented in the following table (Table 1), with a majority abundance for the natural melatonin and AFMK of 233.1 -> 174.1 and 265.1 -> 177.1 respectively.

**Table 1:** Relative isotopic abundances for melatonin, ^13^C melatonin (upper part) and AFMK,^13^C AFMK (bottom part)

Transitions	Natural melatonin	^13^C melatonin
233.1 → 174,1	0.8818127	0.007222551
234.1 → 175.1	0.109686398	0.885270578
235.1 → 176.1	0.008060553	0.100505643
236.1 → 177.1	0.000440349	0.007001228

Transitions	Natural AFMK	^13^C AFMK
265.1 → 178.1	0.889342277	0.009532049
266.1 → 179.1	0.101342851	0.890700889
267.1 → 180.1	0.008903015	0.091847488
268.1 → 181.1	0.000578242	0.007919574

On the other hand, isotopic abundance of its majority transition for melatonin and AFMK 234.1 -> 175.1 and 266.1 -> 179.1 respectively should provide values very close to those presented in Table 1.

**Figure 1:**
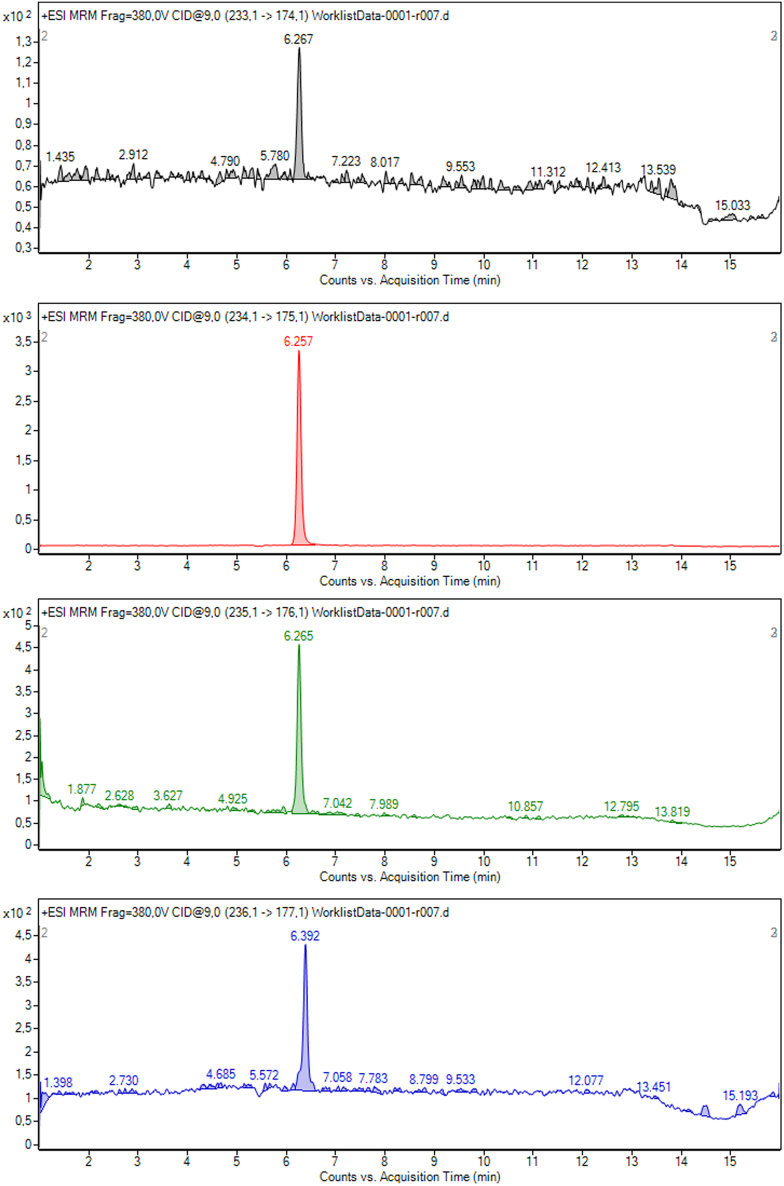
^13^C melatonin 1 ppb standard chromatography spectrum representative of its transitions

**Figure 2:**
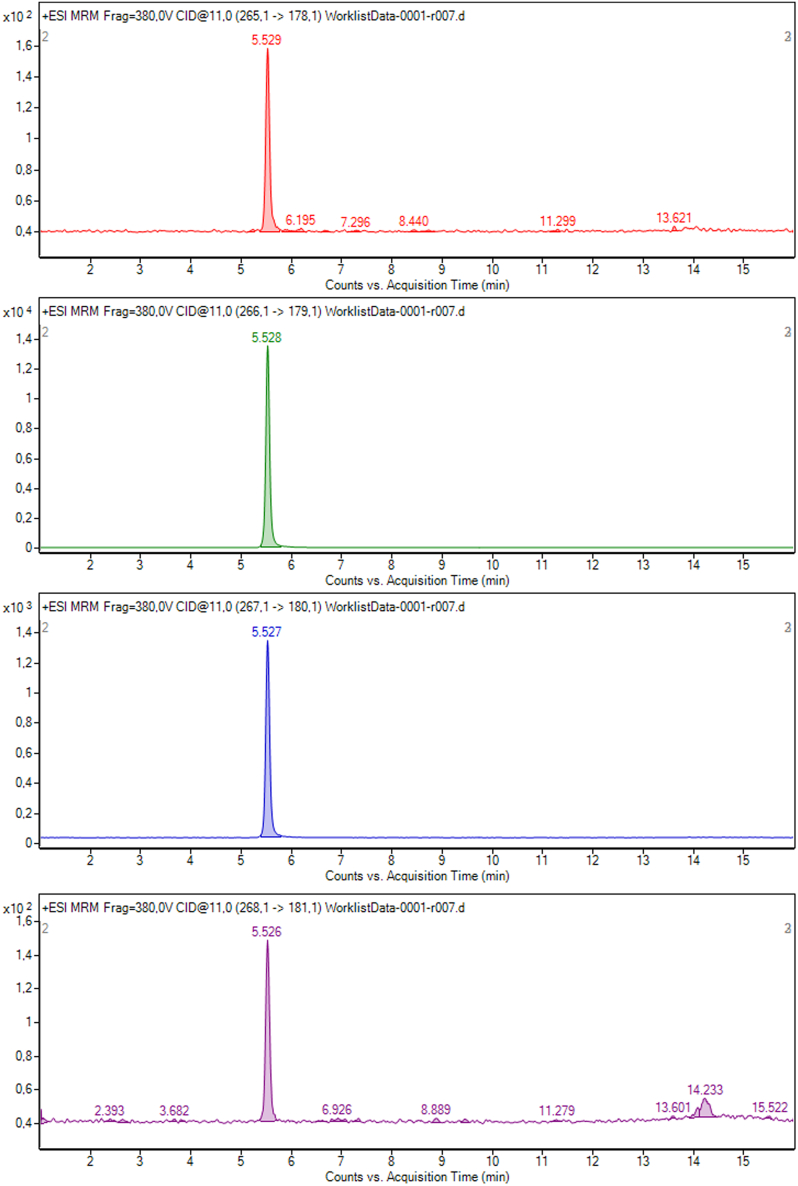
^13^C AFMK 50 ppb standard chromatography spectrum representative of its transitions

**Figure 3:**
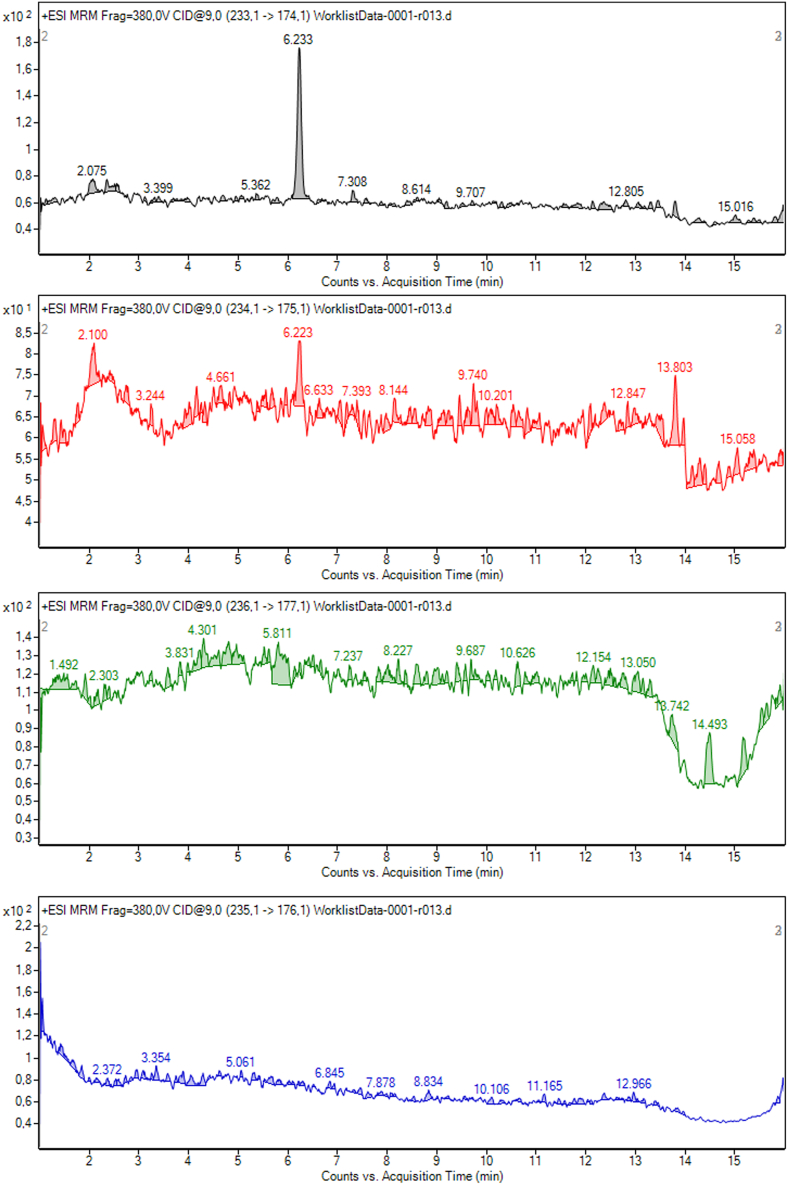
Melatonin chromatography CSF sample spectrum representative of its transitions

**Figure 4:**
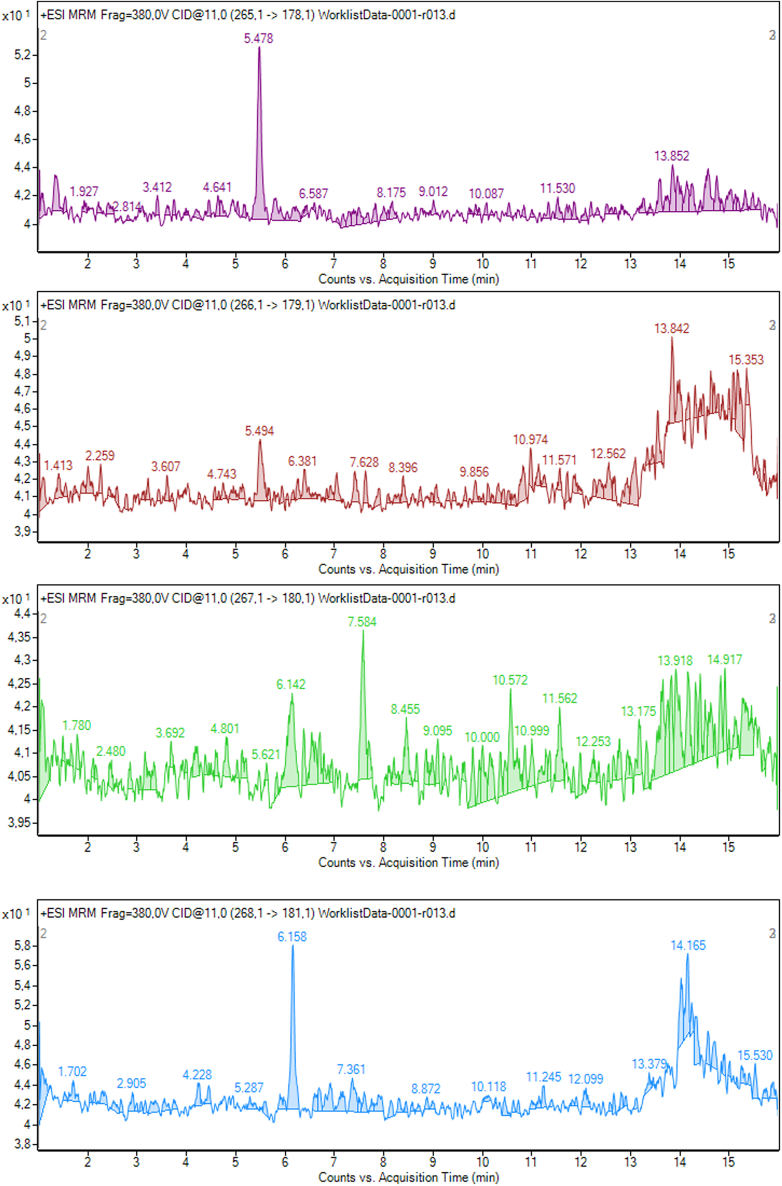
AFMK CSF sample chromatography spectrum representative of its transitions

Once the measurements have been carried out on the LC-MS/MS system, the following chromatographic results (Figures 1, 2, 3, and 4) should be obtained for each of the transitions used in the protocol.

Regarding the expected concentrations in CSF samples, further details of the values obtained in a total of 46 and 48 measurements for melatonin and AFMK refer to Artime et al.^10^

Melatonin and AFMK concentration range is summarized in Table 2.

**Table 2:** Expected melatonin and AFMK range in CSF samples

	Melatonin (pg/ml)	AFMK (pg/mL)
CSF	5–200	10–2500

## Quantification and statistical analysis

This section includes a representative template for the analysis of CSF samples that can be used as a model to perform the final calculation of melatonin and AFMK concentrations in the samples, using both the isotopic dilution standardization protocol (Template quantifications and statistical analysis ID) and the internal standard protocol using 5-methoxytryptofol (Template quantifications and statistical analysis 5-MT).

### Isotopic dilution (Data S2: Template quantifications and statistical analysis ID)

First, the areas of the different calculated transitions are summed, and each of them is divided by the total sum, determining the experimental relative isotopic abundance in the sample.

The molar fraction of the natural compound and the one enriched in ^13^C is then determined by means of a linear estimation calculation between the experimental abundances of each sample and the relative isotopic abundances of the natural and ^13^C-enriched compounds.

Once the molar fraction is known, the formula presented in section step-by-step method details 3- Data processing and concentration calculation is applied, and the result is expressed in ppts (pg/ml).

### 5-Methoxytryptophol (Data S3: Template quantifications and statistical analysis 5-MT)

First, record the value of the transition areas in SIM mode for the determination of 5-MT, melatonin and AFMK and represent the calibration curves for each of the compounds.

To calculate the degree of correction by internal standard 5-MT determinate the experimental concentration of 5-MT in the reconstituted sample and multiply by the mass of the same previously weighed, to obtain the experimental mass of 5-MT in the reconstituted sample.

Once the mass of 5-MT in the reconstituted sample has been determined, divide the value obtained by the theoretical value for a 100% recovery of the 5-MT mass added at the beginning of the procedure to calculate the recuperation factor.

Finally determine the melatonin and AFMK concentration in the reconstituted samples and apply the recuperation factor using the template 'Template quantifications and statistical analysis 5-MT` as a guide.

## Limitations

The potential limitations of the protocol lie in obtaining isotopically labeled standards if chemical synthesis is not possible, and in the chemical lability of the compounds, being imperative to preserve the samples in ultra-freezing prior the analysis. The protocol sensitivity is in the range of pg/ml, so the determination will not be possible for samples with lower contents of both metabolites.

## Troubleshooting

### Problem 1

Signal too low or not detected on the standard curve. Low signals obtained in analyses are typically due to degradation of the standards used, after consecutive freezing and thawing cycles or due to problems in the resolution of the mass spectrometer (related to Step 2: before you begin) (‘Data S4: Freeze and Thaw and LOD-LOQ’).

### Potential solution

Prepare the standard curve solutions again and aliquot, avoiding successive freeze-thaw cycles in subsequent analyses.

Perform mass spectrometer tuning to check and optimize the equipment parameters in the operating ionization mode.

### Problem 2

No detection of melatonin and/or AFMK in the samples (related to Step 2).

### Potential solution

Extract the metabolites using a larger sample volume. In certain samples, the concentration of the metabolites may be low enough to require using a larger number of samples.

Check the mass spectrometer parameters performing a tuning in the working ionization mode.

Extract biological samples again, no detection can occur due to metabolites degradation during extraction and/or storage.

### Problem 3

Low chromatographic retention times (related to Step 2:1).

### Potential solution

Prepare again freshly chromatographic mobile phases.

Monitor and check the pressure; retention times can be modified due to small leaks or drops in pressure.

If the problem persists, replace the chromatographic column. Old or damaged columns dramatically reduce retention times.

## Resource availability

### Lead contact

Further information and requests for resources and reagents should be directed to the lead contact, Dr. Juan C. Mayo (mayojuan@uniovi.es).

### Technical contact

Further information and requests for technical information should be directed to and will be fulfilled by the technical contact, David Hevia-Sánchez (heviadavid@uniovi.es).

### Materials availability

Standard isotopically labeled compound synthesis is described in this protocol and DOI: https://doi.org/10.1016/j.chroma.2021.462752.

This study did not generate other unique reagents.

### Data and code availability

• The original/source data for Table 2. Expected melatonin and AFMK range in CSF samples in this paper is available in https://doi.org/10.1016/j.heliyon.2025.e41841.
• Statistical procedures and quantification examples are included in this protocol as templates named: Template quantifications and statistical analysis 5-MT and Template quantifications and statistical analysis ID.
• The study did not generate new dataset or code.

## Acknowledgments

F.A.-N. was supported by “Ayudas del Programa Severo Ochoa para la formación en investigación y docencia del Principado de Asturias.” This work was supported by grants #PID2019-111418RBI00, from “10.13039/501100011033Agencia Estatal de Investigación, 10.13039/501100004837Ministerio de Ciencia e Innovación, 10.13039/501100006591Gobierno de España” and #ID/2024/00761 from “10.13039/100007801Agencia de Ciencia, Competitividad Empresarial e Innovación del Principado de Asturias.”

We thank the immunology service at the “Hospital Universitario Central de Asturias” for providing the CSF fluids and the clinical data. We would like to extend our sincere gratitude to all participants in the study.

## Author contributions

F.A.-N. and D.H.-S. developed the technical approach. A.G.-G. and P.R.-G. assisted with the chromatographic method and the isotopic enrichment. A.Z. performed the ^13^C isotope synthesis. F.A.-N. and J.C.M. wrote the manuscript, and R.M.S. and J.C.M. revised the final version.

## Declaration of interests

The authors declare no competing interests.

## Supplemental information

Data S1. Melatonin and AFMK ^13^C_1_ nuclear magnetic resonance spectrum, related to preparation one: Organic synthesis of ^13^C_1_ melatonin and ^13^C_1_ AFML

Data S2. Template quantifications and statistical analysis of isotopic dilution, related to expected outcomes (quantification and statistical analysis)

Data S3. Template quantifications and statistical analysis of 5-methoxytryptophol, related to expected outcomes (quantification and statistical analysis)

Data S4. Freeze and thaw and LOD-LOQ, related to troubleshooting
