# Supplementary material for: Protocol for the quantification of melatonin and AFMK in cerebrospinal fluid by LC-MS/MS
Source: STAR Protoc. 2025 Oct 22;6(4):104145. doi: 10.1016/j.xpro.2025.104145 (PMC12590424; doi:10.1016/j.xpro.2025.104145)
Supplement: Data S1. Melatonin and AFMK 13C1 nuclear magnetic resonance spectrum, related to preparation one: Organic synthesis of 13C1 melatonin and 13C1 AFML [file mmc1.pdf]

Data S1: Melatonin and AFMK 13C1 nuclear magnetic resonance spectrum, related to Preparation one: organic synthesis of $^{13}C_1$ Melatonin and $^{13}C_1$ AFML

^{13}C-Melatonin

^{1}H NMR (300 MHz, $CDCl_3$, δ (ppm)): 8.17 (br. s, 1H, NH_{indol}), 7.26 (dd, 1H, J_1=3.0 Hz, J_2=9.0 Hz, H_{arom}), 7.02 (m, 2H, H_{arom}), 6.87 (dd, 1H, J_1=3.0 Hz, J_2=9.0 Hz, H_{arom}), 5.61 (br. s, 1H, NHCO), 3.86 (d, 3H, J=141.1 Hz, $^{13}CH_3O$), 3.58 (q, 2H, J_1=6.0 Hz, J_2=12.0 Hz, CH_2), 2.94 (t, 2H, J= 6.0 Hz, CH_2), 1.92 (s, 3H, CH_3). ^{13}C NMR (75 MHz, $CDCl_3$, δ (ppm)): 170.34, 154.27 (d, J=1.5 Hz), 131.75, 127.93, 123.01, 112.85, 112.64 (d, J=3.0 Hz), 112.23, 100.64 (d, J=4.5 Hz), 56.14, 39.94, 25.47, 23.60. HR MS (TOF MS ES+)(m/z): calculated for $^{13}C^{12}C_{12}H_{16}N_2O_2Na$ $[M+Na]^+$ 256.1143, found 256.1160.

^{13}C-AFMK

^{1}H NMR (300 MHz, $CDCl_3$, δ (ppm)): 11.21 (br. s, 1H, N<u>H</u>CHO), 8.65 (d, 1H, J=9.0 Hz, H_{arom}), 8.43 (d, 1H, J=1.5 Hz, C<u>H</u>O), 7.38 (d, 1H, J=3.0 Hz, H_{arom}), 7.13 (dd, 1H, J_1=3.0 Hz, J_2=9.0 Hz, H_{arom}), 6.28 (br. s, 1H, N<u>H</u>$COCH_3$), 3.84 (d, 3H, J=144.1 Hz, $^{13}CH_3O$), 3.64 (q, 2H, J_1=6.0 Hz, J_2=12.0 Hz, CH_2), 3.27 (t, 2H, J= 6.0 Hz, CH_2), 1.97 (s, 3H, CH_3). ^{13}C NMR (75 MHz, $CDCl_3$, δ (ppm)): 203.44, 170.48, 159.58, 155.07 (d, J=2.3 Hz), 133.33, 123.30, 122.84, 120.89 (d, J=3.8 Hz), 115.70 (d, J=3.8 Hz), 55.87, 39.77, 34.64, 23.41. HR MS (TOF MS ES+)(m/z): calculated for $^{13}C^{12}C_{12}H_{16}N_2O_4Na$ $[M+Na]^+$ 288.1042, found 288.1035.
